# Dynamic changes in cardiac morphology, function, and diffuse myocardial fibrosis duration of diabetes in type 1 and type 2 diabetic mice models using 7.0 T CMR and echocardiography

**DOI:** 10.3389/fendo.2023.1278619

**Published:** 2023-11-08

**Authors:** Hong-Kai Zhang, Chun-Yan Shi, Dong-Ting Liu, Hui-Qiang Gao, Qian-Qian Zhao, Nan Zhang, Lin Yang, Guo-Qi Li, Yue-Li Wang, Yu Du, Qing Li, Kai-Rui Bo, Baiyan Zhuang, Zhan-Ming Fan, Zhong-Hua Sun, Lei Xu

**Affiliations:** ^1^ Department of Radiology, Beijing Anzhen Hospital, Beijing Institute of Heart, Lung, and Vascular Diseases, Capital Medical University, Beijing, China; ^2^ Department of Cardiac Surgery, Beijing Anzhen Hospital, Beijing Institute of Heart, Lung and Vascular Diseases, Capital Medical University, Beijing, China; ^3^ Department of Cardiology, Medical School of Chinese People’s Liberation Army (PLA), Beijing, China; ^4^ Beijing Institute of Heart, Lung, and Vascular Diseases, The Key Laboratory of Remodeling-Related Cardiovascular Diseases, Ministry of Education, Beijing, China; ^5^ Echocardiographic Medical Center, Beijing Anzhen Hospital, Capital Medical University, Beijing, China; ^6^ Department of Cardiology, Clinical Center for Coronary Heart Disease, Beijing Institute of Heart Lung and Blood Vessel Disease, Beijing Anzhen Hospital, Capital Medical University, Beijing, China; ^7^ Discipline of Medical Radiation Science, Curtin Medical School, Perth, WA, Australia

**Keywords:** diabetic cardiomyopathy, cardiac magnetic resonance imaging, feature tracking, cardiac dysfunction, ECV, mouse model

## Abstract

**Background:**

Diabetes mellitus (DM) is associated with an increased risk of cardiovascular disease (CVD). Hence, early detection of cardiac changes by imaging is crucial to reducing cardiovascular complications.

**Purpose:**

Early detection of cardiac changes is crucial to reducing cardiovascular complications. The study aimed to detect the dynamic change in cardiac morphology, function, and diffuse myocardial fibrosis(DMF) associated with T1DM and T2DM mice models.

**Materials and methods:**

4-week-old C57Bl/6J male mice were randomly divided into control (n=30), T1DM (n=30), and T2DM (n=30) groups. A longitudinal study was conducted every 4 weeks using serial 7.0T CMR and echocardiography imaging. Left ventricular ejection fraction (LV EF), tissue tracking parameters, and DMF were measured by cine CMR and extracellular volume fraction (ECV). Global peak circumferential strain (GCPS), peak systolic strain rate (GCPSSR) values were acquired by CMR feature tracking. LV diastolic function parameter (E/E’) was acquired by echocardiography. The correlations between the ECV and cardiac function parameters were assessed by Pearson’s test.

**Results:**

A total of 6 mice were included every 4 weeks in control, T1DM, and T2DM groups for analysis. Compared to control group, an increase was detected in the LV mass and E/E’ ratio, while the values of GCPS, GCPSSR decreased mildly in DM. Compared to T2DM group, GCPS and GCPSSR decreased earlier in T1DM(GCPS 12W,P=0.004; GCPSSR 12W,P=0.04). ECV values showed a significant correlation with GCPS and GCPSSR in DM groups. Moreover, ECV values showed a strong positive correlation with E/E’(T1DM,r=0.757,P<0.001;T2DM, r=0.811,P<0.001).

**Conclusion:**

The combination of ECV and cardiac mechanical parameters provide imaging biomakers for pathophysiology, early diagnosis of cardiac morphology, function and early intervention in diabetic cardiomyopathy in the future.

## Introduction

Diabetes mellitus (DM) is one of the most common chronic metabolic diseases in worldwide, with increasing morbidity. The two major types of DM are type 1 (T1DM) and type 2 (T2DM). T1DM accounts for about 5% of all DM patients, while T2DM is a common disease (1). Previous studies have demonstrated that DM is an independent risk factor for various cardiovascular diseases (CVD), and the morbidity and mortality rate of adverse cardiovascular events in patients with DM is much higher than without DM (2, 3). Glucose and lipid metabolism disorder and inflammatory and oxidative stress are the main pathological mechanisms of abnormal cardiac structure and function in DM, which induce diabetic cardiomyopathy (DCM), leading to diastolic and systolic dysfunction (4, 5).

Echocardiography and cardiac magnetic resonance imaging (CMR) examination are the first choices for the non-invasive evaluation of cardiac changes in DCM (6). Compared to echocardiography, CMR examination can be used to comprehensively evaluate cardiac morphology, function, myocardial tissue characteristics (including myocardial ischemia, infarction, and fibrosis), myocardial microcirculation perfusion, and myocardial viability (7). Also, CMR has the advantages of high soft-tissue resolution, wide imaging field, and better repeatability. Herein, it has been become a one-stop examination method for heart diseases (8).

CMR myocardial strain technique can evaluate the changes in global or local myocardial deformation, quantitatively assess the subtle myocardial movement throughout the cardiac cycle and detect abnormal subtle cardiac function in the early stage of CVD, including hypertension, cardiomyopathies, and ischemic heart disease (9, 10). Several studies have confirmed that the novel technique—CMR feature tracking (CMR-FT) technology is consistent with CMR-tagging technology (the gold standard of CMR strain imaging)—is widely used in clinics and research (10, 11). CMR-FT is based on conventional steady-state free precession (SSFP) sequences with high spatial resolution, without additional sequences, high signal-to-noise ratio, convenient post-processing, and good repeatability (12). It can also be used to detect the early subtle changes in diastolic and systolic function in DCM (5, 6). CMR-T1 mapping technology can quantitatively measure the T1 values of myocardium before and after contrast agent injection and estimate the extracellular volume fraction (ECV) in combination with hematocrit (HCT). The method can also non-invasively detect the degree of diffuse myocardial fibrosis (DMF) in DCM (13). A large number of studies have shown that the degree of DMF is related to cardiac dysfunction (14, 15). Therefore, the changes in DMF and myocardial strain were detected at the early stage of the disease, and early intervention is crucial for DCM prognosis.

In this study, we used T1DM and T2DM mice models, combined with echocardiography, CMR-FT, and CMR-T_1_ mapping techniques to dynamically observe the changes in cardiac morphology, dysfunction, and tissue characteristics at multiple time points.

## Materials and methods

The schematic of the study and CMRI scan protocol is shown in **Supplementary Figure 1**.

### Animals and experimental models

All the mice were housed under 12/12 h light/dark cycles in a standard mouse feeding room maintained at constant temperature at 22°C and 60–70% humidity. Diet and water were allowed freely. The experimental procedures were approved by the Ethics Committee of Laboratory Animals at the Capital Medical University of Beijing, China.

A total of 90 C57Bl/6J male mice (4-week-old) were enrolled in this study and randomly divided into three groups: control (n=30), T1DM (n=30), and T2DM (n=30). The STZ (Sigma–Aldrich, Budapest, Hungary) was diluted in citric acid buffer (pH=4.5; 10 mg/mL) and used to induce the DM model. The control and T1DM groups were fed a normal diet (ND; containing 10% kcal fat D12450B; Research Diets, New Brunswick, NJ, USA), while the T2DM group was fed a high-fat diet (HFD) (containing 60% kcal fat, D12492, Research Diet) to induce insulin resistance. After 4 weeks, mice in the T1DM group were injected intraperitoneally multiple times with low-dose streptozotocin (MLD-STZ) at 60 mg/kg in citric acid buffer for 5 consecutive days (16). Mice in the T2DM group were administered a single intraperitoneal injection of high-dose STZ (SHD-STZ) at 100 mg/kg in the citric acid buffer (17). The control group mice were injected an equivalent volume of citric acid buffer. Blood glucose was measured 5 days after STZ injection. Mice with hyperglycemia (3-h fasting blood glucose levels ≥13.9 mmol/L) were defined as diabetic (16, 17). The 3-h fasting blood glucose was measured from a drop of blood collected from the caudal vein by a digital glucose meter (ACCU-CHEK^®^ Performa, Mannheim Germany). In the control and T1DM groups, a normal diet was fed continuously, and the T2DM group was fed HFD. The experimental procedures were performed for 4–24 weeks every 4 weeks after the successful establishment of the models (about 24 weeks of diabetes duration; **Supplementary Figure 1A**).

### Echocardiography

echocardiography was conducted with a high-resolution Ultrasound cardiovascular system (VEVO 2100, VisualSonics, Toronto, Canada). The 15–20 MHz linear transducer was used to acquire cardiac images. 1-2% Isoflurane anesthesia (in 100% oxygen) was inhaled by mice. Then, the animals were placed in a supine position on a heating board (maintained at 37°C). The cardiac images were analyzed on the VEVO LAB Version-3.1.1 ultrasound workstation. The parameters related to diastolic function were measured: early diastolic peak (E), the late peak (A) velocities, and the ratio of E/A were examined based on the mitral valve inflow pattern. Also, the early diastolic mitral annulus peak velocity (E’), the late diastolic mitral annulus peak velocity (A’), and the ratio of E/E’ and E’/A’ were measured by tissue Doppler. Also, left ventricular (LV) mass and LV mass (corrected) were measured using M-mode. LV mass corrected(mg) = LV MASS × 0.8SV (μL), SV = LVEDV – LVESV); LV mass = 1.04 × [(LVAWd + LVIDd + LVPWd)^3^ – LVIDd^3^]All parameters were averaged over three cardiac cycles (16).

### Magnetic resonance imaging (MRI)

Water and food are avoided for 8–12 h before MRI scanning. The mice were anesthetized with 2% isoflurane and maintained by 1% isoflurane concentration. Then, the animals were imaged using a 7.0 T MRI system and a 4-channel phased-array mouse heart coil (VARIAN, CA, USA). In addition, the tail blood was collected to acquire hematocrit (HCT). The protocol of CMRI is illustrated in **Supplementary Figure 1B**. Pre- and post-T1 mapping, Late-gadolinium enhancement (LGE) images were acquired in the prone position. First, pre-T1 mapping was collected, and then, 0.5 mmol/kg Gd-DTPA contrast agent (Bayer Phamar AG) was injected intraperitoneally. LGE-MRI was performed after 25 min using a multislice inversion recovery sequence. Cine CMRI was acquired as dynamic images in about 7-8 slices in the true short-axis orientation, which covered the whole LV through electrocardiograph and respiratory gating (16). Then, post-T1 mapping was performed 30 min after gadolinium-diethylenetriamine penta acetic acid (Gd-DTPA) injection using a 3-slice GRE Look–Locker inversion recovery sequence from basal to apical segments in the true short-axis images at the end-diastole. The cine MRI imaging parameters were as follows: field of view, 25.6 × 25.6 mm, data matrix, 128× 128, slice thickness,1 mm; echo time, 1.43 ms; time of repetition, 4.6 ms; excitation pulse,17.5°. The Pre- and Post-T1 mapping parameters were as follows: field of view, 25.6×25.6 mm; data matrix, 128×128; slice thickness, 1 mm; echo time (TE), 3.3 ms; time of repetition (TR), 6.5 ms; excitation pulse,15°; flip angle, 20°; inversion times, 45 ms. The LGE-MRI imaging parameters were as follows: field of view, 25.6 × 25.6 mm, data matrix, 128× 128, echo time, 1.1ms; time of repetition, 3.1ms; flip angle, 90°.

The T1 mapping parameters were manually analyzed in a blinded manner using VnmrJ4.0 (VARIAN) post-processing software. A region of interest was defined in the blood pool and LV myocardium to evaluate the pre- and post-contrast T1 values from the basal to apical slices. The ECV was calculated as follows: ECV=(1−hematocrit)×(Δ R1 myocardium/Δ R1 blood), R1 = 1/T1 (13, 14). CMR cardiac function and CMR-FT images were analyzed using the commercial software (cvi^42^, Circle Cardiovascular Imaging Inc., Calgary, AB, Canada). The short-axis cine LV images were used to assess the LV volume, ejection fraction (EF), and two-dimensional (2D) circumferential and radial deformation parameters. The epicardial and endocardial contours were identified, manually outlined, corrected, and automatically propagated in end-systole and end-diastole for LV volume calculation (ESV and EDSV, respectively). Moreover, a 2D incompressible deformable model of the myocardium with individual image slices (SA acquisitions) over the cardiac cycle between the epicardial and endocardial contours was generated by interpolating the tracked boundaries in the largest phase (end-diastolic). The global peak circumferential strain (GCPS), peak systolic strain rate (GCPSSR), peak diastolic strain rate (GCPDSR), global peak radial strain (GRPS), peak systolic strain rate (GRPSSR), and diastolic strain rate (GRPDSR) were acquired by a 2D algorithm. In the end-diastole, the maximum LV wall thickness was measured in 17 LV segment cine images. The areas of LGE within the myocardium were searched actively.

### Histopathological analysis

Histological validation was performed to match each CMR T1 mapping technique to measure the diffuse myocardial fibrosis in DM mice. Three out of six mice were sacrificed every 4 weeks in the control, T1DM, and T2DM groups. The heart weight and tibia length were measured, and the ratio of heart weight/tibia length was calculated. After CMR and echocardiography imaging scans at every time point, animals were euthanized by isoflurane anesthesia. A solution of 10% formalin (Cellstor, CellPath, Newton, UK) was used to fix the heart. Subsequently, 5-μm-thin myocardial slices were subjected to conventional histology (hematoxylin-eosin (H&E) and Sirius-Red) staining. Sirius Red staining was used to measure collagen deposition and calculate collagen volume fraction (CVF). The images were analyzed using NIS-Elements 4.10 software (Nikon, Japan).

### Statistical analysis

Six mice were included every 4 weeks in the control and T1DM groups for statistical analysis in echocardiography and MRI. Shapiro–Wilk test was used to evaluate the distribution of continuous variables. Data are presented as mean ± standard deviation. Homogeneity of variance was tested by Brown–Forsythe and Bartlett tests. The differences between any two of the multiple groups were analyzed by one-way analysis of variance (ANOVA) in control,T1DM and T2DM at multiple time points. The differences between control and T1DM/T2DM or between T1DM and T2DM groups at the same timepoint were analyzed using Student’s t test.The correlation between the two variables was assessed using Pearson’s test. The data of CMR-FT parameters from 36 random mice were assessed using repeated measurements for reproducibility. The intraclass correlation coefficient (ICC) was used as a measure of intra- and interobserver reproducibility. For all comparisons, P<0.05 was considered statistically significant. SPSS 22.0 (IBM, New York, USA) and GraphPad Prism version 6.0 (GraphPad Software, San Diego, CA, USA) were used for statistical analyses and drawing figures.

## Results

### Animal models and general information

#### Bodyweight and blood glucose levels

We dynamically measured the body weights and 3-h no-fasting blood glucose levels in control, T1DM, and T2DM groups (**Supplementary Figure 2**). The average body weight decreased in the T1DM group but increased in the T2DM group compared to the control group duration of the experiment (**Supplementary Figure 2A**). Moreover, the blood glucose levels increased in both T1DM and T2DM groups (**Supplementary Figure 2B**).

#### Cardiac morphology and function from MRI and echocardiography

**Figure 1:**
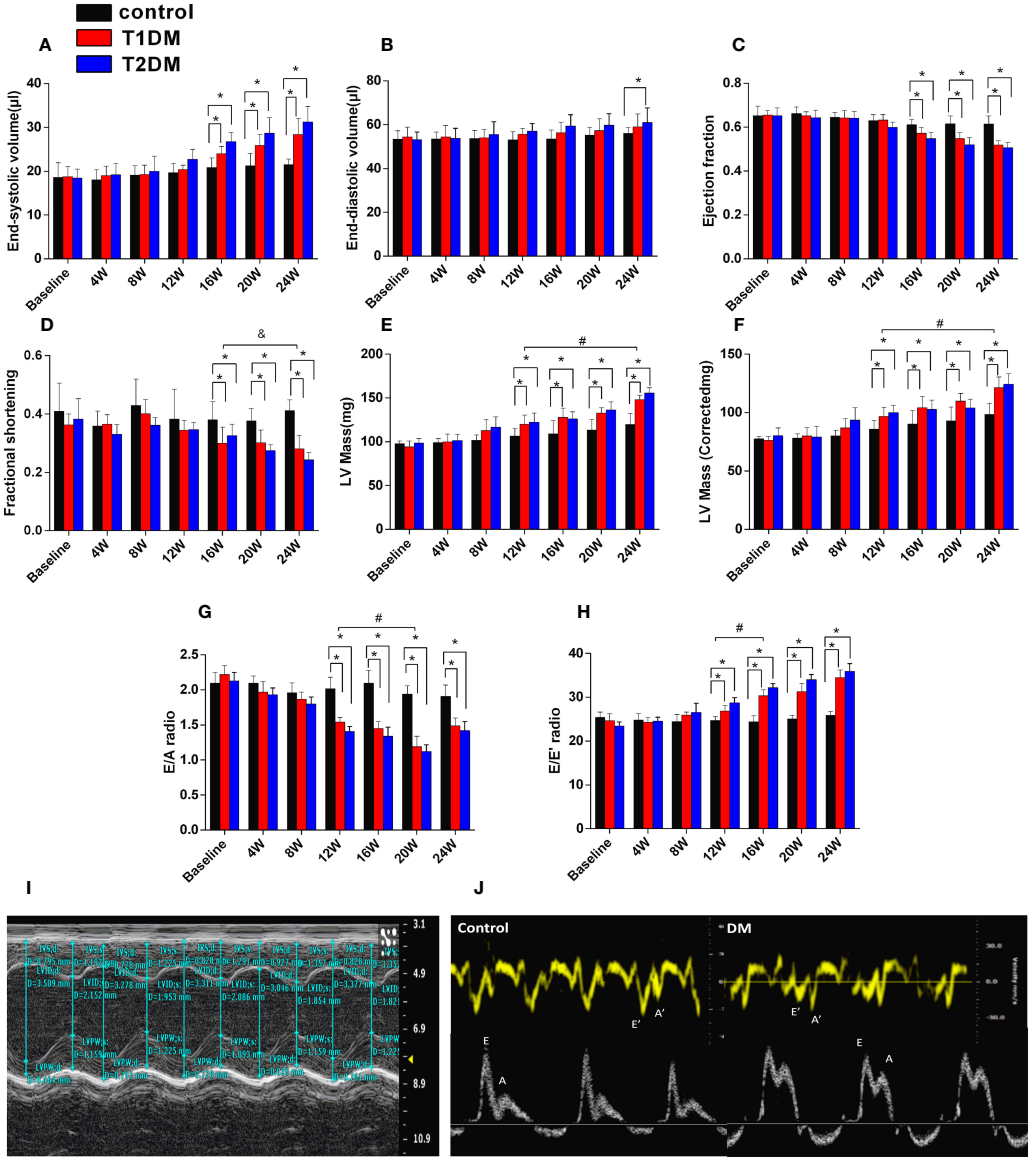
Cardiac structure and function in control, T1DM, and T2DM groups from the baseline to 24 weeks. **(A)** End-systolic volume (ESV); **(B)** end-diastolic volume (EDV); **(C)** ejection fraction (EF); **(D)** fractional shortening (FS). **(E)** LV mass; **(F)** LV mass (corrected). **(G)** E/A radio and **(H)** E/E’ radio; **(I)** LV mass and LV mass (corrected) and **(J)** early diastolic peak **(E)**, the late peak **(A)** velocities, early diastolic mitral annulus peak velocity (E’), the late diastolic mitral annulus peak velocity (A’), acquired by M-mode echocardiography and tissue Doppler imaging (TDI). Data are presented as mean ± standard deviation at the same time point. (t-test) and any two of the multiple groups’ comparison (ANOVA). (n=5 or 6). *P<0.05 vs. control mice; #P<0.05 vs. DM 12W mice; &P<0.05 DM 24 vs. DM 16W mice.

The morphology and function of the LV were assessed by 7.0T CMR-cine and echocardiography-mitral valve inflow pattern and tissue Doppler in T1DM, T2DM, and control groups (**Table 1**, **Figure 1**). Next, we observed a gradual decrease in E wave, E’ wave, E/A radio, and E’/A’ and an increase in A wave, A’ wave, and the ratio of E/E’ from 8–24 weeks. This effect indicated that the diastolic function had deteriorated gradually in the duration of DM in T1DM and T2DM groups. Also, T2DM showed more severe diastolic dysfunction than the T1DM group at the same point from 12–20 weeks (**Table 1**), indicating a gradual increase in the LV volume values from 8–24 weeks (**Figures 1A, B**).

Table 1Basic characteristic and echocardiographic parameters of the control, T1DM, and T2DM groups from baseline to 24 weeks.

**Table:** 

Time (weeks)	Baseline			4			8			12		
Groups	Control	T1DM	T2DM	Control	T1DM	T2DM	Control	T1DM	T2DM	Control	T1DM	T2DM
**HR (bpm)**	392 ± 32	389 ± 36	392 ± 37	394 ± 36	382 ± 37	421 ± 61	390 ± 33	383 ± 26	438 ± 47	396 ± 29	404 ± 30	384 ± 43
**Resp (bpm)**	35 ± 3	33 ± 4	32 ± 5	37 ± 4	33 ± 2	38 ± 5	38 ± 4	36 ± 4	37 ± 6	38 ± 3	37 ± 3	36 ± 5
**E’ wave (mm/s)**	28.07 ± 2.46	28.32 ± 1.89	29.68 ± 1.86	28.8 ± 1.79	28.77 ± 0.78	28.18 ± 1.12	29.25 ± 2.23	26.88 ± 1*	25.8 ± 1.45*	28.72 ± 0.99	24.57 ± 0.87*	23.45 ± 1*
**A’ wave (mm/s)**	18.58 ± 1.72	17.9 ± 1.38	20.67 ± 0.86	19.22 ± 1.64	18.18 ± 0.94	19.1 ± 0.88	18.05 ± 1.73	18.03 ± 0.87	19.45 ± 1.15	18.23 ± 0.73	21.97 ± 2.15*	22.63 ± 1.99*
**E’/A’ (radio)**	1.52 ± 0.14	1.59 ± 0.1	1.44 ± 0.07	1.51 ± 0.14	1.59 ± 0.11	1.48 ± 0.12	1.62 ± 0.08	1.49 ± 0.07	1.28 ± 0.16*	1.58 ± 0.04	1.13 ± 0.12*	1.04 ± 0.05*
**A wave (mm/s)**	339.17 ± 28.2	314.67 ± 12.15	327.64 ± 26.21	342.75 ± 19.78	357.15 ± 26.06	358.67 ± 19.29	365.59 ± 23.64	369.02 ± 19.19	381.49 ± 45.92	353.38 ± 27.41	429.16 ± 26.71*	479.84 ± 34.82*
**E wave (mm/s)**	709.97 ± 26.25	696.4 ± 17.04	695.92 ± 27.66	710.64 ± 14.63	699.08 ± 18.83	691.55 ± 26.27	713.24 ± 11.84	697.56 ± 19.43	684.71 ± 55.13	709.95 ± 16.55	659.14 ± 11.65*	672.98 ± 34.73*
**E/A (ratio)**	2.1 ± 0.15	2.22 ± 0.13	2.13 ± 0.12	2.08 ± 0.11	1.97 ± 0.15	1.93 ± 0.1	1.96 ± 0.14	1.87 ± 0.1	1.81 ± 0.14	2.02 ± 0.16	1.54 ± 0.07*	1.41 ± 0.07* $
**E/E’(ratio)**	25.39 ± 1.24	24.67 ± 1.53	23.48 ± 0.87	24.75 ± 1.52	24.32 ± 1.03	24.55 ± 0.89	24.48 ± 1.58	25.96 ± 0.66	26.57 ± 2.09	24.74 ± 0.88	26.86 ± 1.24*	28.71 ± 1.2*$
**LV mass (mg)**	97.9 ± 3.09	94.28 ± 6.42	98.54 ± 5.12	99.07 ± 4.54	100.09 ± 8.84	101.48 ± 7.18	101.71 ± 6.16	112.82 ± 12.56	116.63 ± 11.85	106.35 ± 8.65	120.09 ± 10.42*	122.22 ± 10.65*
Time (weeks)	Baseline			16			20			24

**Table:** 

Groups	Control	T1DM	T2DM	Control	T1DM	T2DM	Control	T1DM	T2DM	Control	T1DM	T2DM
**HR (bpm)**	392 ± 32	389 ± 36	392 ± 37	401 ± 26	389 ± 35	407 ± 47	403 ± 45	400 ± 38	371 ± 38	395 ± 41	365 ± 36	389 ± 31
**Resp (bpm)**	35 ± 3	33 ± 4	32 ± 5	37 ± 3	37 ± 6	36 ± 4	35 ± 4	38 ± 4	35 ± 4	35 ± 3	34 ± 3	37 ± 5
**E’ wave (mm/s)**	28.07 ± 2.46	28.32 ± 1.89	29.68 ± 1.86	28.47 ± 2.12	21.23 ± 1.38*	19.95 ± 1.18*	27.83 ± 1.35	18.9 ± 1.56*	16.92 ± 1.02*&	27.08 ± 0.84	18.02 ± 1.6*	16.92 ± 1.09*
**A’ wave (mm/s)**	18.58 ± 1.72	17.9 ± 1.38	20.67 ± 0.86	18.1 ± 1.24	20.43 ± 1.87	22.33 ± 1.61*	18.6 ± 0.92	22.68 ± 2.64	23.42 ± 2.02*	17.82 ± 1.66	23.9 ± 1.7	24.13 ± 1.42*
**E’/A’ (ratio)**	1.52 ± 0.14	1.59 ± 0.1	1.44 ± 0.07	1.58 ± 0.2	1.05 ± 0.11*	0.9 ± 0.11*&	1.5 ± 0.1	0.84 ± 0.05*	0.72 ± 0.03*&	1.53 ± 0.13	0.75 ± 0.03*	0.7 ± 0.07*
**A wave (mm/s)**	339.17 ± 28.2	314.67 ± 12.15	327.64 ± 26.21	332.24 ± 32.39	447.07 ± 39.53*	483.34 ± 55.79*	360.57 ± 23.87	500.79 ± 40.48*#	515.08 ± 40.33*	370.36 ± 37.92	416.96 ± 39.71*	430.69 ± 36.3*
**E wave (mm/s)**	709.97 ± 26.25	696.4 ± 17.04	695.92 ± 27.66	693.05 ± 21	643.29 ± 21.63*	641.57 ± 24.78*	696.63 ± 18.25	589.9 ± 39.48*#	574.74 ± 33.65*#	701.7 ± 21.96	620.46 ± 37.48*	606.14 ± 11.33*
**E/A (ratio)**	2.1 ± 0.15	2.22 ± 0.13	2.13 ± 0.12	2.1 ± 0.18	1.45 ± 0.1*	1.34 ± 0.13*	1.94 ± 0.12	1.19 ± 0.15*#	1.12 ± 0.1*#	1.91 ± 0.16	1.49 ± 0.11*	1.42 ± 0.13*
**E/E’ (ratio)**	25.39 ± 1.24	24.67 ± 1.53	23.48 ± 0.87	24.43 ± 1.38	30.36 ± 1.35*#	32.2 ± 0.94*#$	25.06 ± 0.83	31.3 ± 1.9*	34 ± 1.19*$	25.92 ± 0.87	34.53 ± 1.73*	35.93 ± 1.8*
**LV mass (mg)**	97.9 ± 3.09	94.28 ± 6.42	98.54 ± 5.12	109.2 ± 15.04	127.91 ± 10.44*	126.02 ± 8.31*	113.37 ± 12.04	132.69 ± 6.2*	136.65 ± 9.0*	119.77 ± 12.47	148.25 ± 4.81*#	155.6 ± 6.2*# $

Parameters of the control, T1DM, and T2DM groups from baseline to 24 weeks.

Heart rate (HR), respiration (Resp), E wave: early diastolic peak wave, A wave: late peak (A) wave velocities in mitral valve inflow pattern, the early diastolic mitral annulus peak velocity (E’) wave, the late diastolic mitral annulus peak velocity (A’) wave by tissue Doppler imaging, the ratio of E/A, E’/A’ and E/E’. Data are presented as mean ± standard deviation, * and $, P<0.05 vs. control group and T2DM vs. T1DM at the same time point. (t-test) and any two of the multiple groups’ comparison (ANOVA).

#P<0.05 vs. DM 12-week-old mice (A wave, E wave, E/A ratio, LV mass).

&P<0.05 vs. DM 8-week-old mice (E’ wave and E’/A’ ratio).

**Figure 2:**
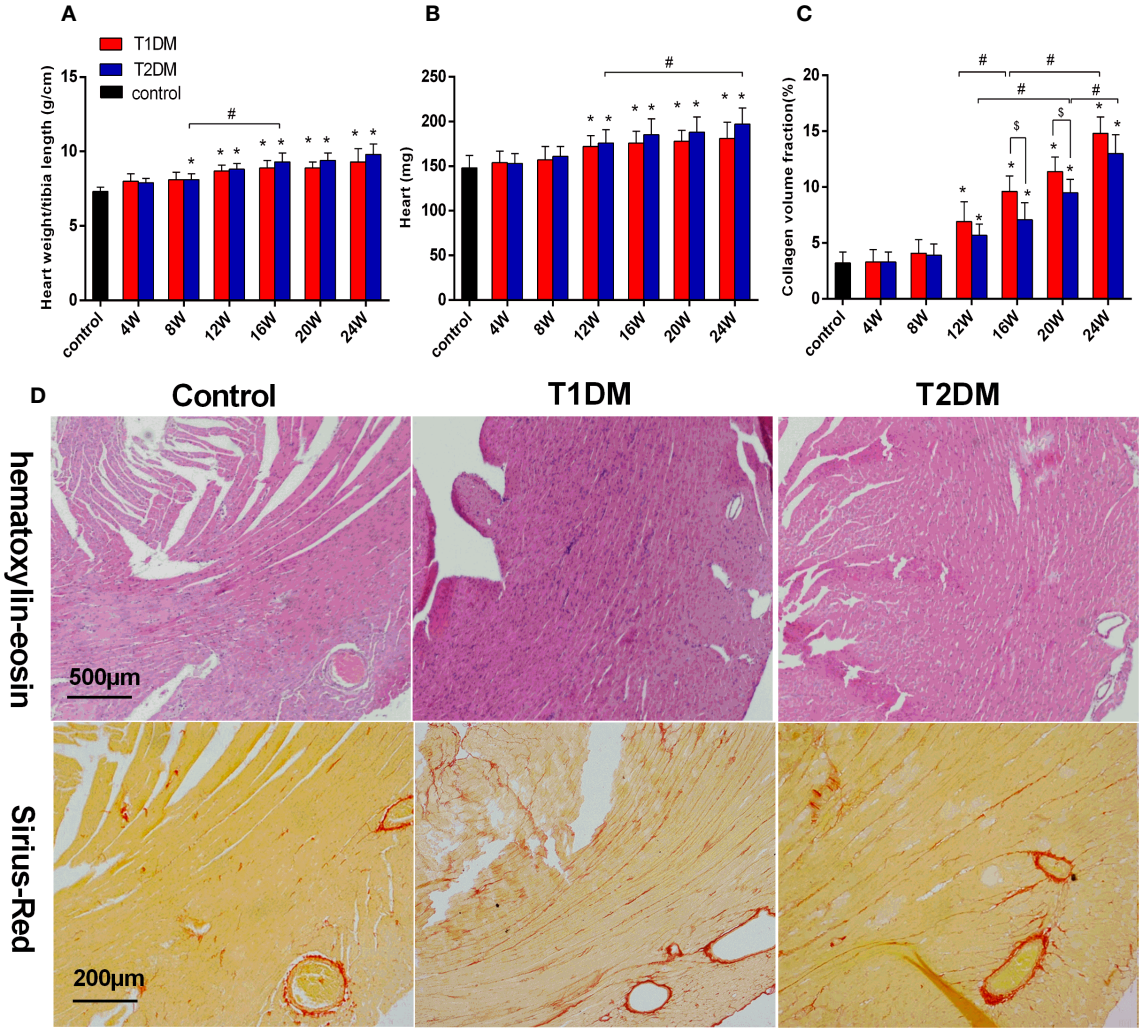
Bar plot of the heart weight **(A)** and the ratio of heart weight/tibia length **(B)** and collagen volume fraction **(C)** in the control, T1DM, and T2DM groups. **(D)** H&E staining: Compared to the control group, the sections of the heart shows edema, histiocytic proliferation, and hemorrhage in T1DM and T2DM groups at 12 weeks. Sirius-Red staining (red=fibrosis, orange=myocardial fibers): Compared to the control group, diffuse interstitial fibrosis became increasingly serious in both T1DM and T2DM groups at 12 weeks. Data are presented as mean ± standard deviation (n=3); * and $, P<0.05 vs. control group and T2DM vs. T1DM at the same time point. #P<0.05 vs. between the DM groups at different time points. (t-test) and any two of the multiple groups’ comparison (ANOVA) were used.

Additionally, EF progressively decreased in T1DM and T2DM from 4–24 weeks, while EF was maintained within the normal range (**Figure 1C**). The LV mass and LV mass (corrected) increased from 4–24 weeks in both T1DM and T2DM groups (**Figures 1E, F**), which might be induced by cardiac hypertrophy and confirmed by the increased ratio of the heart weight to tibia length (**Figure 2A**).

**Figure 3:**
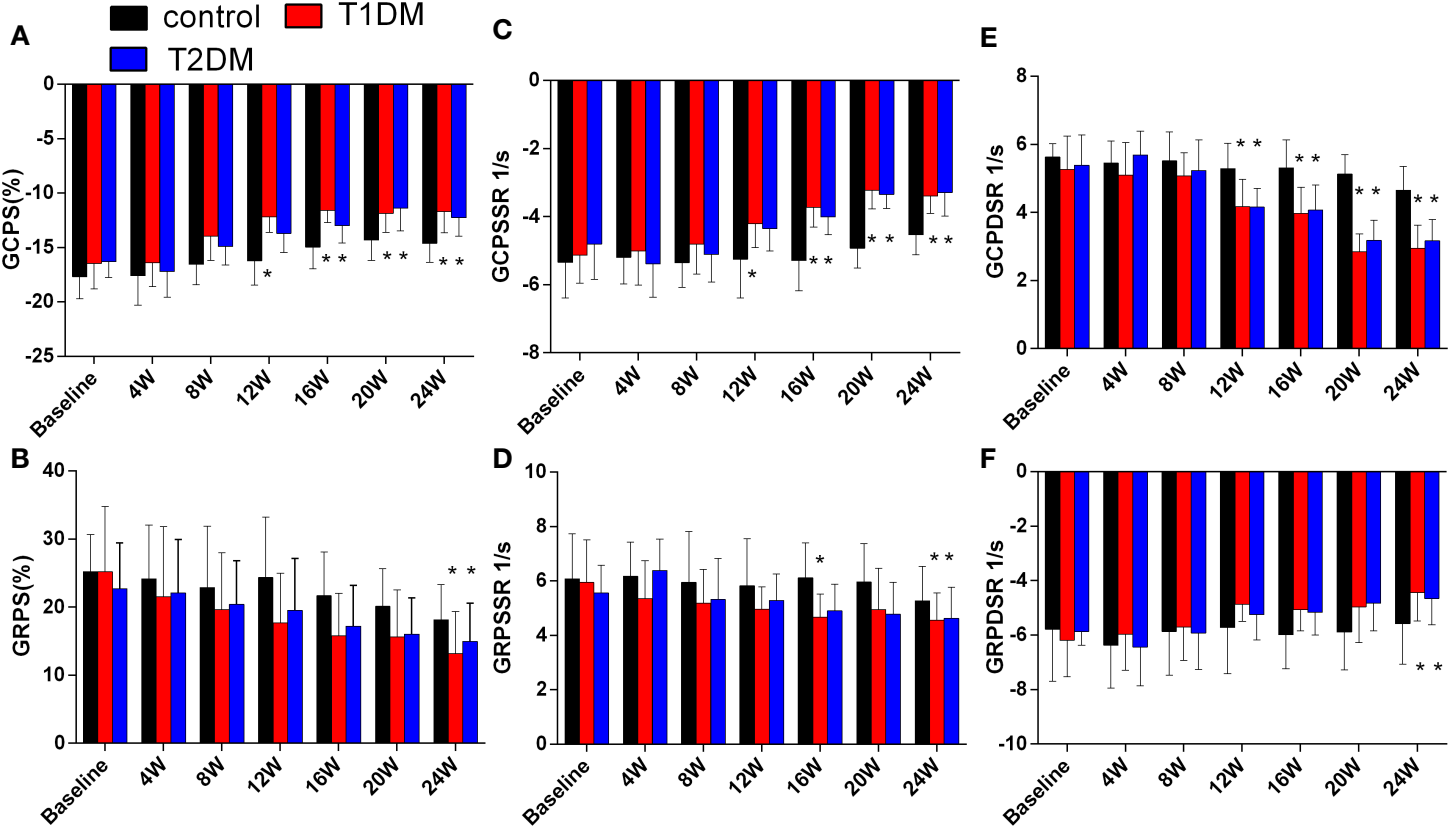
Cardiac strain and strain rate in the control, T1DM, and T2DM groups from baseline to 24 weeks. **(A)** Global peak circumferential strain (GCPS); **(B)** Global peak radial strain (GRPS); **(C)** Global peak circumferential systolic strain rate (GCPSSR); **(D)** Global peak radial systolic strain rate (GRPSSR); **(E)** Global peak circumferential diastolic strain rate (GCPDSR); **(F)** Global peak radial diastolic strain rate (GRPDSR). Data are presented as mean ± standard deviation at the same time point. (t-test) and any two of the multiple groups’ comparison (ANOVA). (n=6). *P<0.05 vs. control mice.

Compared to the control groups, a gradual decrease was observed in the absolute value of GCPS, GRPS, GRPSSR, GCPSSR, GRPDSR, and GCPDSR in the T1DM and T2DM groups (**Supplementary Table 1**, **Figure 3**, **Supplementary Figure 3**). Compared to the T2DM group, GCPS and GCPSSR decreased earlier in the T1DM group (T1DM 12W: GCPS -12.18 ± 1.41 vs. -16.21 ± 2.23, P=0.004; GCPSSR -4.21 ± 0.69 vs. -5.25 ± 1.13, P=0.04; T2DM 16W: GCPS -12.99 ± 1.61 vs. -14.97 ± 1.97, P=0.005; GCPSSR -4.01 ± 0.52 vs. -3.72 ± 0.59, P=0.02) (**Figures 3A, C**). Moreover, the GCPDSR value showed a significant decrease at 12 weeks in T1DM and T2DM (T1DM 4.17 ± 0.80 vs. Control 5.29 ± 0.74, P=0.030; T2DM 4.16 ± 0.55 vs. 5.29 ± 0.74, P=0.014) (**Supplementary Table 1**, **Figure 3E**).

#### Cardiac magnetic resonance T mapping

**Figure 4:**
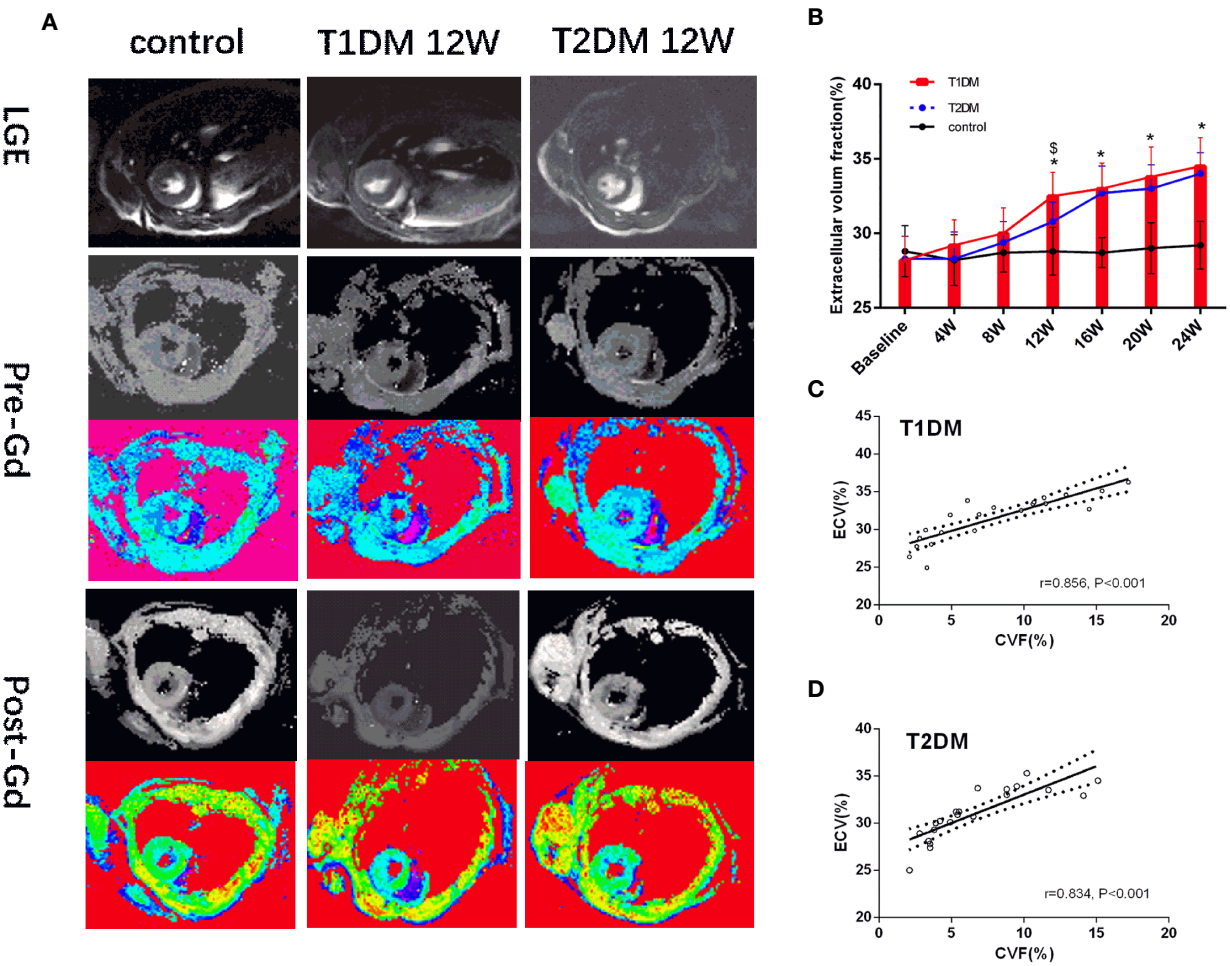
**(A)** Pre- and post-contrast T1 maps from control, T1DM, and T2DM group at 12 weeks. Color scale bar shows T1 values from 0–2000 ms. **(B)** A bar plot for myocardial ECV parameters in the control, T1DM, and T2DM groups from 4–24 weeks. **(C, D)** Correlation between extracellular volume fraction (ECV) and collagen volume fraction (CVF) in T1DM and T2DM. *and $, P<0.05 vs. control group and T2DM vs. T1DM at the same time point.

Currently, there is no evidence of focal late contrast enhancement (LGE) in any DM groups (**Figure 4A**). The CMR ECV parameters are shown in **Figure 4B**. The ECV differed significantly between the DM and control groups (T1DM: 32.5 ± 1.6% vs. 28.1 ± 1.8%, P=0.002; T2DM: 31.1 ± 1.2% vs. control 28.1 ± 1.8%, P=0.044) at 12 weeks (**Figure 4B**). Compared to the T2DM groups, the ECV value was higher in the T1DM group at 12 weeks (T1DM 12 weeks vs. T2DM 12 weeks, P<0.048).

#### Histological analysis of myocardial fibrosis and myocardial hypertrophy

The heart weight and the ratio of the heart weight/tibia length were significantly increased in both the T1DM and T2DM groups compared to the control group (heart weight/tibia length: T1DM 12 weeks vs. control, P<0.001; T2DM 8 weeks vs. control, P=0.036; T2DM 8 weeks vs. T2DM 16 weeks, P=0.009; heart weight: T1DM 12 weeks vs. control, P<0.013; T2DM 8 weeks vs. control, P=0.011; T2DM 12 weeks vs. T2DM 24 weeks, P=0.045) (**Figures 2A, B**). Heart sections stained with Sirius-red showed a progressive induction of extensive fibrosis throughout the myocardium in both DM groups. At 12 weeks, the CVF values in both DM groups showed a significant difference from the control group (T1DM: 6.9 ± 1.8% vs. 3.3 ± 1.1%, P<0.01; T2DM: 5.1 ± 1.1% vs. 3.3 ± 1.1%, P<0.01) (**Figure 2C**). Additionally, CVF values increased gradually in the course of diabetes (T1DM 16W: 9.6 ± 1.4% vs. T1DM 12W, P<0.046: T1DM 24W: 14.8 ± 1.5% vs. T1DM 16W, P<0.001; T2DM 20W: 9.5 ± 1.2% vs. T2DM 12W, P<0.001; T2DM 24W: 13.0 ± 1.7% vs. T2DM 20W, P=0.013). Compared to the T2DM groups, the CVF values were higher in the T1DM group at 16 and 20 weeks (T1DM 16W vs. T2DM 16W: 7.1 ± 1.5%, P<0.026; T1DM 20W: 11.4 ± 1.3% vs. T2DM 20W, P<0.035). The histological results of H&E and Sirius Red staining of typical mice in the control, T1DM, and T2DM groups at 12 weeks are displayed (**Figure 2D**).

#### Correlation between the ECV and CVF

A strong positive correlation was detected between ECV and CVF (T1DM: r=0.856, P<0.001; T2DM: r=0.834, P<0.001) (**Figures 4C, D**). Additonally, there was a moderate positive correlation between pre-contrast myocardium T1 and CVF(T1DM: r=0.557, P<0.001; T2DM: r=0.538, P<0.001) in **Supplementary Figures 4B, C**.

#### Correlation between cardiac systolic dysfunction indicators and ECV

**Figure 5:**
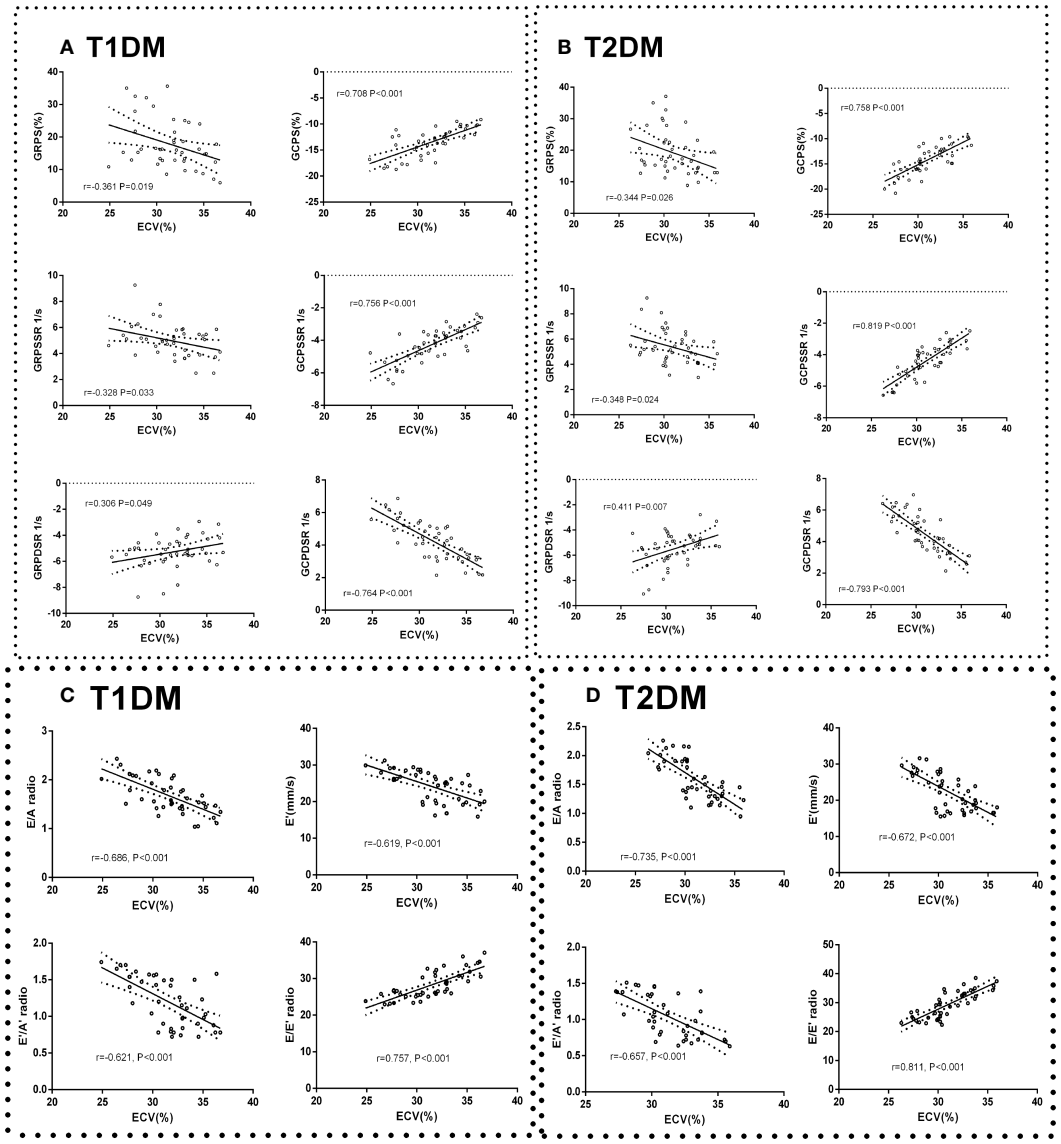
Correlation between cardiac systolic dysfunction indicators and ECV in control, T1DM, and T2DM groups at each time point. Correlation between GCPS, GRPS, GRPSSR, GCPSSR GRPDSR, GCPDSR, and ECV in T1DM **(A)** and T2DM **(B)**. Correlation between E/A, E’, E’/A’, E/E’, and ECV in T1DM **(C)** and T2DM **(D)**.

Global peak radial strain (GRPS), global peak radial systolic strain rate (GRPSSR) and global peak radial diastolic strain rate (GRPDSR) had a general correlation with ECV in T1DM and T2DM (GRPS: T1DM, r=-0.361, P=0.091; T2DM, r=-0.344, P=0.026; GRPSSR: T1DM, r=-0.328, P=0.033; T2DM, r=-0.348, P=0.024; GRPDSR: T1DM, r=0.306, P=0.049; T2DM, r=0.411, P=0.007) (**Figures 5A, B**). Global peak circumferential strain (GCPS), global peak circumferential systolic strain rate (GCPSSR) and global peak circumferential diastolic strain rate (GCPDSR) had a significant correlation with ECV in T1DM and T2DM (GCPS: T1DM, r=708, P<0.001; T2DM, r=0.758, P<0.001; GCPSSR: T1DM, r=0.756, P<0.001; T2DM, r=0.819, P<0.001; GCPDSR: T1DM, r=-0.764, P<0.001; T2DM, r=-0.793, P<0.001) (**Figures 5A, B**).

#### Correlation between cardiac diastolic dysfunction indicators and ECV

The ratio of E/A, E’/A’, and E’ had a moderate negative correlation with ECV (E/A: T1DM, r=-0.686, P<0.001; T2DM, r=-0.735, P<0.001; E’: T1DM r=-0.619, P<0.001; T2DM, r=-0.672, P<0.001; E’/A’: T1DM, r=-0.621, P<0.001; T2DM r=-0.657, P<0.001) (**Figure 5C**). The E/E’ ratio was strongly positively correlated with ECV (T1DM r=0.757, P<0.001; T2DM r=0.811, P<0.001) (**Figure 5D**).

#### Intra− and interobserver variability of CMR-FT parameters

The ICC values were high in GCPSSR (intraobserver and interobserver: 0.932 and 0.949, respectively) and GCPDSR (intraobserver and interobserver: 0.925 and 0.933, respectively). GCPS showed almost no difference between intra- and interobserver variability (0.910 and 0.908, respectively). The ICC values were low in GRPS (0.826 and 0.811), GRPSSR (0.824 and 0.835), and GRPDSR (0.846 and 0.831).

## Discussion

In this study, we performed dynamic evaluation by the combination of histologic verification, CMR T1 mapping (ECV), myocardial mechanics (strain and strain rate), and echocardiography parameters (E/A and E/E’) at multiple time points in T1DM and T2DM mice models. The present study aimed to monitor the dynamic changes in cardiac morphology, function, myocardial mechanics, and diffuse myocardial fibrosis in T1DM and T2DM mice models and found that: (a) CMR feature tracking technology measurements are sensitive in the detection of DCM associated with cardiac dysfunction both systolic and diastolic function. On the other hand, ECV value increased gradually with diabetic progression and could detect the decrease in early cardiac dysfunction. Notably, at the 12-week time point, ECV exhibited a statistically significant difference between the DM and control groups. (b) The changes in ECV were detected based on the markers of diastolic dysfunction, and T2DM showed more severe diastolic dysfunction than the T1DM group at the same point from 12–20 weeks. (c) The ECV was significantly correlated with the E/E’ ratio, CVF, GCPS, GCPSSR, and GCPDSR and could continuously assess the degree of DMF and cardiac dysfunction during diabetes.

The diabetic mouse model showed several advantages. First, the mice model of MLD-STZ-induced T1DM and the SHD-STZ-induced T2DM were widely used in diabetic animal studies that present the phenotypes of DCM (17, 18). According to the type of DM, DCM is closely related to specific blood glucose levels and body weight changes. T1DM is linked to weight loss and insulin deficiency, while T2DM is related to insulin resistance and obesity (19). Moreover, there are multiple advantages of high consistency, good repeatability, disease progression, and reduced effects of concomitant disease, including hypertension and coronary heart disease (20). In line with these findings, our results showed elevated blood glucose levels in both T1DM and T2DM groups during the course of DM; however, body weight was lower in T1DM but increased in T2DM. Second, using ultra-high field 7.0 T CMR and echocardiography imaging *in vivo*, we could longitudinally observe DMF, myocardial remodeling, and cardiac dysfunction within several months at multiple timepoints rather than years or decades in humans, and the histological correlation with CMR markers of LV fibrosis, including ECV was verified. In addition, it facilitated the ECV correlation with markers of myocardial mechanics and cardiac dysfunction in DCM mice. Finally, our DM mice model provided insights into the DMF and cardiac dysfunction based on longitudinal imaging evaluation.

Although transthoracic echocardiography is the most common imaging method to diagnose DCM (especially diastolic dysfunction) with the advantages of cost-efficiency and no radiation (21). CMR has additional advantages, such as better spatial resolution and less dependence on image quality compared to echocardiography (22). Especially, CMR can quantitatively detect the tissue characteristics of myocardial histology, such as myocardial fibrosis, edema, hemorrhage, and abnormal material deposits (23–25). Thus, it is a reference technique for the assessment of cardiac morphology and function, such as LV volume, mass, and ejection fraction (22). Additionally, CMR-FT also can be used to detect the subtle changes in diastolic and systolic function in the early stage of DCM (9). Herein, compared to the control group, a gradual increase was observed in ESV from 16–24 weeks in T1DM and T2DM. Also, LV mass, heart weight beginning at 12 weeks, and the ratio of heart weight/tibia length at 8 weeks increased, suggesting that myocardial hypertrophy occurred in the early stage of diabetes. Furthermore, the heart weight and heart weight/tibia length values did not differ significantly between T1DM and T2DM mice; however, T2DM was higher than T1DM, which might be related to their pathophysiological mechanisms (25–27). Additionally, compared to the control group, we observed a gradual decrease in E wave, E’ wave, E/A ratio, E’/A’ increase, A’ wave, and the ratio of E’/E in the duration of DM. EF progressively decreased in the DM group, but remained within the normal range. Interestingly, the EF value showed significant differences at 16 weeks, whereas E/A and E/E’ ratio occurred at 12 weeks in both T1DM and T2DM compared to the control group. Furthermore, compared to the control groups, the CMR-FT parameters decreased gradually in the absolute value of GCPS, GRPS, GRPSSR, GCPSSR GRPDSR, and GCPDSR in the T1DM and T2DM groups. Compared to the T2DM group, GCPS and GCPSSR decreased earlier at 12 weeks in the T1DM group, while the GCPDSR value showed a significant decrease at 12 weeks in the T1DM and T2DM groups. These findings indicated that T1DM has an earlier onset of reduced systolic function than T2DM. Also, diastolic dysfunction appeared earlier than systolic dysfunction, which deteriorated gradually during DM, which was consistent with the previous study (28) and Mátyás et al (18). Several studies have shown that E/E’ is the most reliable and stable indicator of diastolic dysfunction (29–31). In the present study, the T2DM group showed a more severe diastolic dysfunction than T1DM, and diastolic stiffness was impaired due to the changes in the E/E’ ratio at the same time points from 12–20 weeks. According to the clinical and animal studies, this finding may be related to the pathophysiological mechanism of T1DM and T2DM (11, 27, 32), and cardiac stiffness was common in T2DM (26). T2DM was more likely to exhibit EF preserved cardiac dysfunction than T1DM (18).

Due to respiratory and heart rate effects, the characterization of cardiac performance is challenging in DM mice (33). A rapid heart rate and high respiratory rate may affect the image quality of cardiac tissue features (T1 mapping). In recent years, CMR T1 mapping has been commonly used as a non-invasive technique to detect DMF (34); especially, ECV reflects the changes in the myocardial extracellular matrix (35). It is a reliable imaging biomarker to quantitatively assess the degree of DMF and dynamically assess the characteristics of early-stage DCM (14, 16). Also, ECV is seldom affected by factors such as the acquisition time, HCT, contrast agent wash in and wash out in disease conditions, and renal excretion rate (36). Several studies have shown that myocardial fibrosis is detected through ECV in DM with normal LVEF (16, 37). In the current study, the ECV value was gradually increased in the duration of diabetes, which is consistent with the results of previous studies (14, 16). Compared to the control group, ECV showed a significant difference at 12 weeks in both T1DM and T2DM, which was in line with CFV. In addition, T1DM had a higher ECV value than T2DM at 12 weeks. This phenomenon indicated that T1DM had more severe DMF and earlier changes in systolic cardiac dysfunction than T2DM. Furthermore, our study showed that the ECV value was strongly correlated with CVF; thus, high ECV values mean severe myocardial fibrosis. Nonetheless, no LGE regions were observed in the myocardium in both T1DM and T2DM mice, indicating that DMF is the predominant change in the early stage of DCM without obvious stenosis or obstruction of the large epicardial vessels, leading to replacement fibrosis caused by ischemic myocardial injury. Some studies showed that LGE regions were detected in DM patients (2, 38); some concomitant diseases, including valvular heart, myocardial metabolic, or no-diagnosed coronary heart diseases, might lead to focal myocardial fibrosis (39). The present study also showed that the ECV value was higher than the CVF maybe because the CVF value only reflects the extracellular collagen fiber content, while the ECV value can also exhibit mucus matrix, lipid, and necrosis components outside the cell (26, 34). In the early stage of DCM, the pathophysiological changes were oxidative stress, inflammatory reaction, edema, fatty degeneration of the heart, lipid deposition, and mild myocardial fibrosis (26). Thus, the combination of these pathological processes altered cardiac morphology and function.

Furthermore, our study showed a strong correlation between ECV and the E/E’ ratio,while E/A, E’/A’, and E’ showed moderate but significant correlations. Thus, the E/E’ ratio value was deemed a precise imaging biomaker to reflect diastolic dysfunction and one of the most reproducible and reliable values (29). Additionally, ECV had a strong correlation with GCPS, GCPSSR, and GCPSSR in the DM groups (especially T2DM), indicating that higher ECV values are linked to severe cardiac dysfunction. The early stage of DCM usually exhibits systemic metabolic disorders, mitochondrial dysfunction, oxidative stress, and immunoregulation disorders (26). Typically, myocardial fibrosis and collagen deposition are the earliest pathological changes in the DCM (6). These can lead to increased LV stiffness, resulting in LV dysfunction (4). Thus, ECV could be used to assess the comprehensive pathological status (myocardial fibrosis) and cardiac function changes during DM. To the best of our knowledge, this is the first study to use T1DM and T2DM mice models, combined with echocardiography, 7.0T CMR-FT, and CMR-T1 mapping techniques to dynamically observe the changes in cardiac morphology, dysfunction, and tissue characteristics at multiple time points. Subsequently, in DCM, the changes in DMF, myocardial mechanics, and cardiac diastolic and systolic dysfunction were observed.

### Study limitations

Nevertheless, the present study has some limitations. Firstly, the animal sample size may be small, although it is based on statistical calculations (Power Analysis and Sample Size software). Secondly, due to the limitations of scanning conditions, we did not obtain a long-axis image to measure the longitudinal strain and strain rate. Although longitudinal strain is the most robust parameter in human studies, short-axis images were feasible tools in various experimental studies and pathological states. Finally, only DM mice were utilized in this study. DM patients often have multiple diseases, including coronary heart disease and hypertension. Thus, a large sample size is essential for human studies in the future.

## Conclusion

This is the study on dynamic monitoring of early diffuse myocardial fibrosis at multiple time points using 7.0 T CMR T1 mapping-derived ECV parameters and CMR feature tracking technology-derived cardiac mechanical parameters combined with echocardiography-derived diastolic dysfunction parameters in T1DM and T2DM mice models. The characteristic differences were compared between T1DM and T2DM experimental animal models. Moreover, the myocardial strain, strain rate, and ECV parameter values were useful tools to evaluate the dynamic changes in cardiac morphology and function, as well as myocardial mechanics and fibrosis in T1DM and T2DM mice models. The ECV value could be applied to assess the degree of early DMF in DM groups at 12 weeks. ECV and cardiac mechanical parameters can provide imaging scores for the pathophysiology, early clinical diagnosis, and the efficacy of medication treatment in T1DM and T2DM.

## Data availability statement

The original contributions presented in the study are included in the article/**Supplementary Material**. Further inquiries can be directed to the corresponding author.

## Ethics statement

The animal study was approved by The experimental procedures for mice were approved by the Ethics Committee of Laboratory Animals at the Capital Medical University of China (Permission Number: AEEI-2018-020). The study was conducted in accordance with the local legislation and institutional requirements.

## Author contributions

HZ: Conceptualization, Data curation, Formal Analysis, Investigation, Methodology, Software, Validation, Writing – original draft. CS: Conceptualization, Data curation, Software, Writing – original draft, Writing – review & editing. DL: Data curation, Funding acquisition, Methodology, Software, Writing – original draft. HG: Data curation, Investigation, Methodology, Validation, Writing – review & editing. QZ: Data curation, Formal Analysis, Methodology, Resources, Writing – review & editing. NZ: Data curation, Methodology, Supervision, Visualization, Writing – review & editing. LY: Data curation, Investigation, Project administration, Writing – review & editing. GL: Conceptualization, Formal Analysis, Software, Writing – review & editing. YW: Resources, Software, Validation, Writing – review & editing. YD: Data curation, Investigation, Resources, Software, Writing – review & editing. QL: Investigation, Software, Writing – original draft. KB: Data curation, Investigation, Validation, Writing – original draft. BZ: Data curation, Formal Analysis, Software, Writing – original draft. ZF: Conceptualization, Formal Analysis, Funding acquisition, Investigation, Project administration, Resources, Writing – original draft, Writing – review & editing. ZS: Conceptualization, Formal Analysis, Investigation, Supervision, Validation, Writing – review & editing. LX: Conceptualization, Funding acquisition, Investigation, Project administration, Resources, Visualization, Writing – review & editing.

## Supplementary material

Supplementary Table 1 MRI parameters of the control, T1DM, and T2DM groups from baseline to 24 weeks post-successful models. Parameters of the control, T1DM, and T2DM groups from baseline to 24 weeks. End-diastole volume (EDV), end-systole volume (ESV), ejection fraction (EF), global peak circumferential strain (GCPS), global peak radial strain (GRPS), global peak circumferential systolic strain rate (GCPSSR), global peak radial systolic strain rate (GRPSSR), global peak circumferential diastolic strain rate (GCPDSR), global peak radial diastolic strain rate (GRPDSR), and extracellular volume fraction (ECV). Data are presented as mean ± standard deviation, * and $, P<0.05 vs. control group and T2DM vs. T1DM at the same time points. (t-test) and any two of the multiple groups’ comparison (ANOVA).

Supplementary Figure 1 Schematic of the study and CMRI scan protocol. **(A)** STZ was used to make T1DM and T2DM mice models. MRI, echocardiography, and histopathology were performed at multiple time points (baseline,4–24 weeks, every 4 weeks). **(B)** 7.0T MRI system from VARIAN. MRI acquisition protocol for control, T1DM, and T2DM groups. STZ, streptozotocin; HFD, high-fat diet; MLD-STZ, low-dose streptozotocin; SHD-STZ, high-dose STZ; IR-LL, inversion recovery Look-Locker, LGE-MRI, late-gadolinium enhancement magnetic resonance imaging.

Supplementary Figure 2 Comparison of average weight **(A)** and blood glucose levels **(B)** in control, T1DM, and T2DM groups from 0–24 weeks. At each time point, 6 mice were randomly chosen from each group, and the mean ± standard deviation was plotted as dots and bars, respectively. P<0.001 vs. control mice (t-test).

Supplementary Figure 3 Layout of CMR-FT analysis referring to GCPS, GRPS, GRPSSR, GCPSSR GRPDSR, GCPDSR in the control, T1DM, and T2DM groups at 16 weeks.

Supplementary Figure 4 A bar plot for pre-myocardial T1 in control,T1DM and T2DM groups from baseline to 24 weeks **(A)**. Correlation between pre-myocardial T1 and CVF in T1DM **(B)** and T2DM **(C)** groups at each time point. A bar plot for blood insulin levels in control,T1DM and T2DM groups **(D)**.The mean ± standard deviation was plotted as dots and bars, respectively. * and $, P<0.05 vs. control group and T2DM vs. T1DM at the same time points.
