# Supplementary material for: Dynamic changes in cardiac morphology, function, and diffuse myocardial fibrosis duration of diabetes in type 1 and type 2 diabetic mice models using 7.0 T CMR and echocardiography
Source: Front Endocrinol (Lausanne). 2023 Nov 8;14:1278619. doi: 10.3389/fendo.2023.1278619 (PMC10663371; doi:10.3389/fendo.2023.1278619)
Supplement: Supplementary Table 1 — MRI parameters of the control, T1DM, and T2DM groups from baseline to 24 weeks post-successful models. Parameters of the control, T1DM, and T2DM groups from baseline to 24 weeks. End-diastole volume (EDV), end-systole volume (ESV), ejection fraction (EF), global peak circumferential strain (GCPS), global peak radial strain (GRPS), global peak circumferential systolic strain rate (GCPSSR), global peak radial systolic strain rate (GRPSSR), global peak circumferential diastolic strain rate (GCPDSR), global peak radial diastolic strain rate (GRPDSR), and extracellular volume fraction (ECV). Data are presented as mean ± standard deviation, * and $, P<0.05 vs. control group and T2DM vs. T1DM at the same time points. (t-test) and any two of the multiple groups’ comparison (ANOVA). [file Table_1.docx]

**Supplementary Table 1A. MRI parameters of the control, T1DM, and T2DM groups from baseline to 24 weeks post-successful models**

| **Time (weeks)** | **Baseline** | | | **4** | | | **8** | | | **12** | | |
| --- | --- | --- | --- | --- | --- | --- | --- | --- | --- | --- | --- | --- |
| **Groups** | **Control** | **T1DM** | **T2DM** | **Control** | **T1DM** | **T2DM** | **Control** | **T1DM** | **T2DM** | **Control** | **T1DM** | **T2DM** |
| **EDV (μL)** | 53.5±3.75 | 54.44±4.43 | 53.2±3.41 | 52.72±3.04 | 53.56±3.15 | 53.81±4.5 | 53.63±3.66 | 53.96±3.9 | 55.55±5.79 | 53.19±3.72 | 55.63±2.67 | 57.09±3.49 |
| **ESV (μL)** | 18.58±3.34 | 18.79±2.29 | 18.44±2.09 | 18.06±2.25 | 18.06±2.25 | 19.2±2.61 | 19.11±2.13 | 19.29±2.1 | 20.02±3.38 | 19.68±2.14 | 20.38±1.01 | 22.03±2.19 |
| **EF** | 0.65±0.04 | 0.66±0.02 | 0.65±0.03 | 0.66±0.04 | 0.66±0.03 | 0.64±0.03 | 0.64±0.02 | 0.64±0.03 | 0.64±0.03 | 0.63±0.03 | 0.63±0.02 | 0.61±0.02 |
| **LV mass (mg)** | 98.11±4.15 | 95.32±5.94 | 98.89±5.45 | 99.78±4.88 | 100.78±7.67 | 103.57±6.95 | 103.67±7.08 | 110.76±13.05 | 115.91±12.33 | 107.88±9.24 | 121.12±10.13* | 124.34±11.78* |
| **GCPS (%)** | -17.68±2.02 | -16.47±2.34 | -16.30±1.44 | -17.60±2.27 | -16.40±2.18 | -17.19±2.36 | -16.56±1.86 | -13.94±2.24 | -14.91±1.69 | -16.21±2.23 | -12.18±1.41* | -13.69±1.78 |
| **GRPS (%)** | 25.22±5.48 | 25.20±9.59 | 22.73±6.72 | 24.16±7.91 | 21.55±10.2 | 22.12±7.78 | 21.38±7.49 | 19.64±8.38 | 20.41±6.43 | 20.17±5.64 | 17.69±7.30 | 19.55±7.64 |
| **GRPSSR 1/s** | 6.08±1.65 | 5.95±1.55 | 5.16±1.02 | 6.17±1.26 | 5.35±1.39 | 6.39±1.15 | 5.95±1.87 | 5.18±1.25 | 5.32±1.50 | 5.82±1.73 | 4.96±0.82 | 5.29±0.97 |
| **GCPSSR 1/s** | -5.25±1.13 | -5.13±0.82 | -4.80±1.04 | -5.20±0.78 | -5.00±1.01 | -5.38±0.99 | -5.35±0.73 | -4.80±0.89 | -5.11±0.81 | -5.25±1.13 | -4.21±0.69* | -4.35±0.66 |
| **GRPDSR 1/s** | -5.78±1.91 | -6.19±1.33 | -5.76±0.51 | -6.04±1.51 | -5.97±1.32 | -6.44±1.42 | -5.86±1.61 | -5.70±1.23 | -5.92±1.34 | -5.72±1.69 | -4.87±0.62 | -5.25±0.92 |
| **GCPDSR 1/s** | 5.63±0.39 | 5.26±0.98 | 5.19±0.89 | 5.45±0.65 | 5.10±0.95 | 5.69±0.70 | 5.52±0.84 | 5.07±0.68 | 5.24±0.90 | 5.29±0.74 | 4.17±0.80* | 4.16±0.55* |
| **ECV (%)** | 28.8±1.7 | 28.0±2.1 | 28.3±1.5 | 28.5±2.1 | 29.2±1.7 | 28.3±1.8 | 29.3±1.5 | 30.0±1.7 | 29.4±1.4 | 29.1±1.7 | 32.5±1.6*$ | 30.8±1.3* |

**Supplementary Table 1B. MRI parameters of the control, T1DM, and T2DM groups from baseline to 24 weeks post-successful models**

| **Time (weeks)** | **Baseline** | | | **16** | | | **20** | | | **24** | | |
| --- | --- | --- | --- | --- | --- | --- | --- | --- | --- | --- | --- | --- |
| **Groups** | **Control** | **T1DM** | **T2DM** | **Control** | **T1DM** | **T2DM** | **Control** | **T1DM** | **T2DM** | **Control** | **T1DM** | **T2DM** |
| **EDV (μL)** | 53.5±3.75 | 54.44±4.43 | 53.2±3.41 | 53.59±3.97 | 56.35±4.75 | 59.49±5.16 | 55.22±3.53 | 57.27±5.36 | 59.74±5.29 | 21.48±1.32 | 58.97±6.02* | 63.2±4.51* |
| **ESV (μL)** | 18.58±3.34 | 18.79±2.29 | 18.44±2.09 | 20.86±2.18 | 23.99±1.67* | 26.8±2.02* | 21.28±2.8 | 25.89±2.51* | 28.69±3.54* | 0.62±0.04 | 28.41±3.63* | 31.21±3.62* |
| **EF** | 0.65±0.04 | 0.66±0.02 | 0.65±0.03 | 0.61±0.02 | 0.57±0.03* | 0.55±0.03* | 0.62±0.04 | 0.55±0.03* | 0.52±0.03* | 3.66±0.18 | 0.52±0.02* | 0.51±0.02* |
| **LV mass (mg)** | 98.11±4.15 | 95.32±5.94 | 98.89±5.45 | 111.31±14.72 | 129.11±13.32* | 131.23±10.12* | 114.42±11.56 | 135.46±10.94* | 138.78±11.31* | 120.56±11.44 | 149.51±6.77*# | 157.5±8.1*# $ |
| **GCPS (%)** | -17.68±2.02 | -16.47±2.34 | -16.30±1.44 | -14.97±1.97 | -11.59±1.10* | -12.99±1.61* | -14.30±1.89 | -11.86±1.73* | -11.38±2.08* | -14.61±1.75 | -11.68±1.96* | -12.27±1.69* |
| **GRPS (%)** | 25.22±5.48 | 25.20±9.59 | 22.73±6.72 | 21.02±6.33 | 15.79±6.22 | 17.21±6.01 | 19.11±5.26 | 15.60±6.92 | 16.06±5.33 | 17.64±5.54 | 13.17±6.23* | 14.98±5.60* |
| **GRPSSR 1/s** | 6.08±1.65 | 5.95±1.55 | 5.16±1.02 | 6.11±1.29 | 4.67±0.85* | 4.90±0.98 | 5.96±1.41 | 4.95±1.51 | 4.78±1.17 | 5.27±1.27 | 4.55±1.00* | 4.63±1.14* |
| **GCPSSR 1/s** | -5.25±1.13 | -5.13±0.82 | -4.80±1.04 | -5.29±0.89 | -3.72±0.59* | -4.01±0.52* | -4.93±0.58 | -3.23±0.54* | -3.34±0.42* | -4.53±0.60 | -3.39±0.52* | -3.29±0.69* |
| **GRPDSR 1/s** | -5.78±1.91 | -6.19±1.33 | -5.76±0.51 | -5.98±1.25 | -5.06±0.78 | -5.16±0.83 | -5.89±1.38 | -4.96±1.31 | -4.83±1.01 | -5.58±1.48 | -4.43±1.05* | -4.65±0.97* |
| **GCPDSR 1/s** | 5.63±0.39 | 5.26±0.98 | 5.19±0.89 | 5.31±0.82 | 3.97±0.77* | 4.07±0.73* | 5.13±0.57 | 2.85±0.53* | 3.18±0.59* | 4.66±0.70 | 2.95±0.67* | 3.17±0.62* |
| **ECV** | 28.8±1.7 | 28.0±2.1 | 28.3±1.5 | 28.9±1.4 | 33.0±1.7* | 32.7±1.8* | 27.8±1.5 | 33.8±2.0* | 33.0±1.6* | 29.0±1.9 | 34.5±1.9* | 34.0±1.4* |

Parameters of the control, T1DM, and T2DM groups from baseline to 24 weeks. End-diastole volume (EDV), end-systole volume (ESV), ejection fraction (EF), global peak circumferential strain (GCPS), global peak radial strain (GRPS), global peak circumferential systolic strain rate (GCPSSR), global peak radial systolic strain rate (GRPSSR), global peak circumferential diastolic strain rate (GCPDSR), global peak radial diastolic strain rate (GRPDSR), and extracellular volume fraction (ECV). Data are presented as mean±standard deviation, * and $, P<0.05 *vs*. control group and T2DM *vs*. T1DM at the same time points. (t-test) and any two of the multiple groups’ comparison (ANOVA).
